# 167. Incidence of Acute Kidney Injury with Aminoglycoside Impregnated Foreign Body Implantation

**DOI:** 10.1093/ofid/ofab466.369

**Published:** 2021-12-04

**Authors:** Kelly Royster, Dominic Chan

**Affiliations:** 1 Cheyenne Regional Medical Center, Sherwood, Oregon; 2 Legacy Health, Portland, OR

## Abstract

**Background:**

During orthopedic surgeries, antibiotic impregnated cement is sometimes used to prevent infection. Elution from these cements can lead to systemically detectable levels of aminoglycosides, a known adverse effect of which is nephrotoxicity. The purpose of this study is to determine if the implantation of aminoglycoside impregnated cement is associated with subsequent development of Acute Kidney Injury (AKI).

**Methods:**

A retrospective chart review from 1/1/2018-1/1/2021 was conducted to identify a relationship between aminoglycoside impregnated cement and subsequent development of AKI. Data were extracted from Electronic Health Records (Epic) and SAP Business Objects WebI. All patients with knee or hip arthroplasty or hardware removal procedures conducted at a Legacy Health facility during the specified time frame were included. Patients were excluded from the study if < 2 serum creatinine levels were drawn during that hospitalization, AKI occurred prior to the procedure, or dialysis was required at baseline. The primary outcome was development of AKI, a > 150% increase from baseline serum creatinine according to the Acute Kidney Injury Network (AKIN) criteria. The power level was set to 80% with an alpha level of 0.05. A multiple regression analysis was conducted to control for confounding variables

**Results:**

A total of 2229 patients were included (591 received aminoglycoside cement,1638 did not). Aminoglycoside impregnated cement implantation was not associated with an increased incidence of AKI (1.5% versus 2.3%, P = 0.25). After controlling for covariates, aminoglycoside cement was not associated with development of AKI (adjusted OR 0.68, P = 0.32).

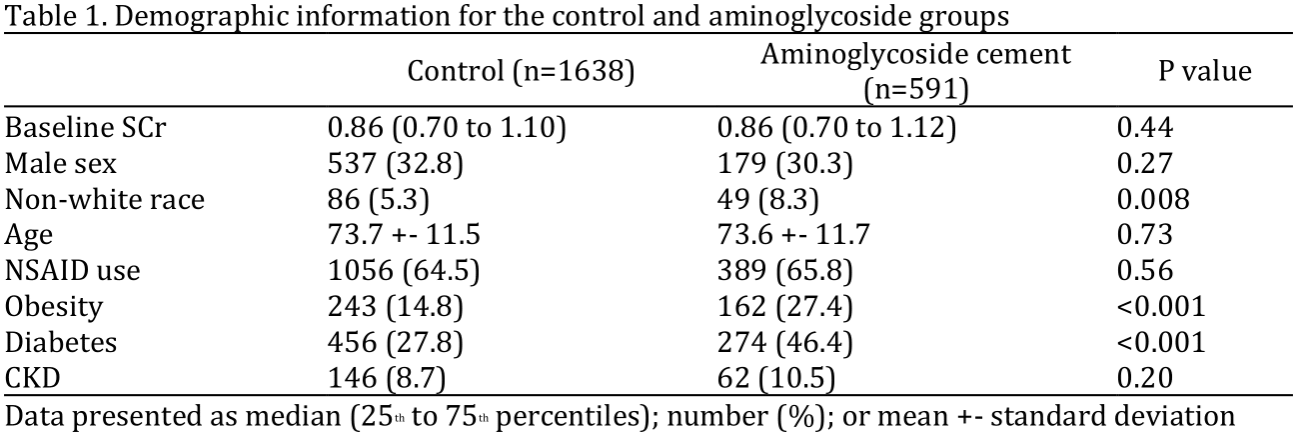

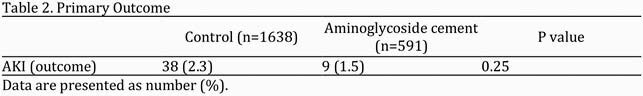

**Conclusion:**

The results of this study suggest aminoglycoside impregnated foreign body implantation was not associated with a greater incidence of AKI development compared to implantation of foreign bodies lacking aminoglycosides. It is possible that development of AKI post-discharge was not identified in patients with uncomplicated procedures due to omission of lab draws once discharged. Patients admitted for longer durations were more likely to have multiple serum creatinine labs drawn during hospitalization, and likely had multiple comorbid conditions or complications, innately biasing and predisposing AKI development.

**Disclosures:**

**All Authors**: No reported disclosures

